# Synthesis of a novel analogue of DPP-4 inhibitor Alogliptin: Introduction of a spirocyclic moiety on the piperidine ring

**DOI:** 10.3762/bjoc.6.71

**Published:** 2010-07-01

**Authors:** Arumugam Kodimuthali, Padala Lakshmi Prasunamba, Manojit Pal

**Affiliations:** 1 New Drug Discovery, R&D Center, Matrix Laboratories Ltd, Anrich Industrial Estate, Bollaram, Jinnaram Mandal, Medak District, Andra Pradesh 502 325, India; 2 Department of Chemistry, University College of Science, Osmania University, Hyderabad 500 007, India; 3 Present address: Institute of Life Science, University of Hyderabad Campus, Gachibowli, Hyderabad 500 046, Andhra Pradesh, India

**Keywords:** Alogliptin, cyclopropyl ring, DPP-4, piperidine

## Abstract

We report the synthesis of a novel analogue of Alogliptin via condensation of two key intermediates one of which is an aminopiperidine derivative bearing a spirocyclic ring on the piperidine moiety. Preparation of the aminopiperidine intermediate was carried out by constructing the cyclopropyl ring prior to assembling the piperidine ring.

## Introduction

**Figure 1:**
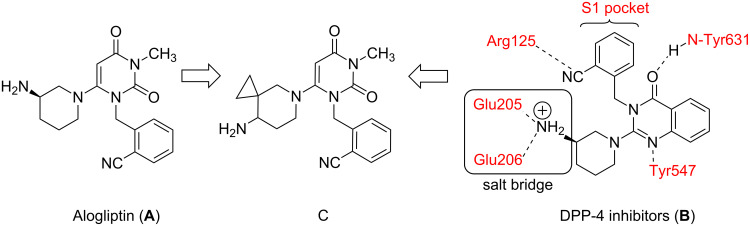
Design of a new DPP-4 inhibitor (**C**) based on Alogliptin (**A**) and other inhibitors (**B**).

Inhibition of dipeptidyl peptidase-4 (DPP-4; CD26; E.C. 3.4.14.5) by small molecules has emerged as one of the key approaches for the treatment of type-2 diabetes [1–5]. DPP-4, a member of the prolyl oligopeptidase family of serine protease, cleaves the *N*-terminal dipeptide from peptides with proline or alanine in the second position. A large number of DPP-4 inhibitors have been reported in the literature [6–7] including NVP-LAF237 (Vildagliptin), MK-0431 (Sitagliptin) and BMS-477118 (Saxagliptin). Presently, Sitagliptin is available for clinical use and Vildagliptin has been launched in Europe only. Several other inhibitors are in various stages of development. For example, Alogliptin or (2-[[6-[(3R)-3-amino-1-piperidinyl]-3,4-dihydro-3-methyl-2,4-dioxo-1(2*H*)-pyrimidinyl]methyl]benzonitrile) (**A**, Figure 1), a potent (IC_50_ < 10 nM) and selective inhibitor (selectivity > 10,000 over DPP-8 and 9) is currently being evaluated in phase 3 clinical trials [8–10]. This compound was identified by replacing the quinazolinone moiety of another inhibitor **B** with a pyrimidine dione (Figure 1) [8]. All these inhibitors that belong to the non-peptidomimetic class contain an aminopiperidinyl moiety which interacts with the DPP-4 enzyme through salt bridges. As part of our ongoing drug discovery program we sought to prepare a novel DPP-4 inhibitor of structure **C** (Figure 1) bearing a spirocyclic ring on the piperidinyl moiety. Our aim was to study the effect of this structurally modified piperidinyl moiety on the interaction of **C** with DPP-4 enzyme. To the best of our knowledge this type of Alogliptin analogue has not yet been explored. Herein we report an efficient synthesis of compound **C** starting from readily available starting materials and reagents.

## Results and Discussion

**Figure 2:**
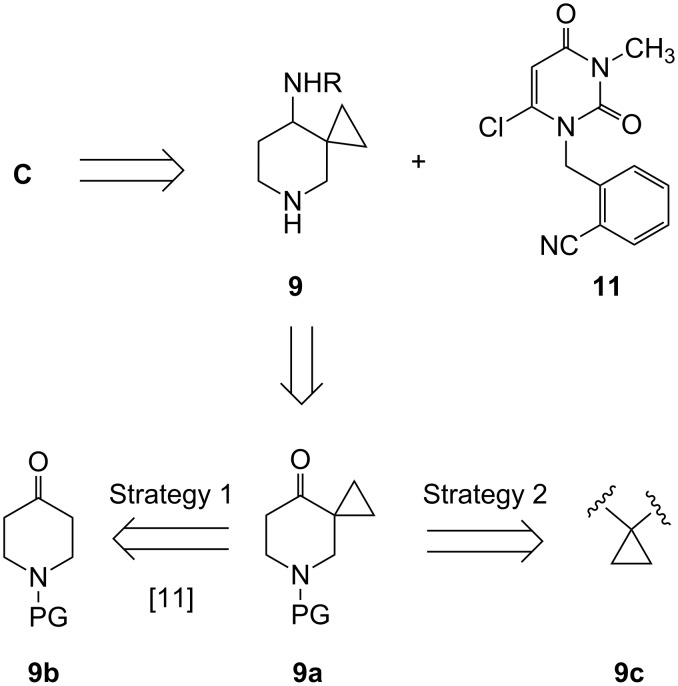
Strategy to prepare compound **C** (PG = protecting group).

**Scheme 1:**
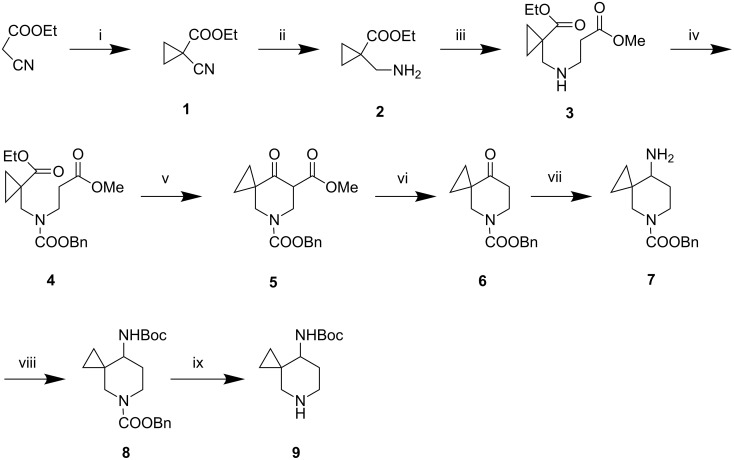
Reagents and conditions: (i) EtONa, BrCH_2_CH_2_Br, EtOH, reflux, 3.5 h (70% yield); (ii) Pd/C, MeOH, H_2_, RT, 4 h (93% yield); (iii) CH_2_=CHCOOMe, THF, RT, 3 h (74% yield); (iv) ClCOOBn, TEA, DCM, 0–5 °C, 3 h (80% yield); (v) NaH, DMF, RT, 7 h (86% yield); (vi) NaCl, DMSO, H_2_O, 110 °C, 7 h (76% yield); (vii) NH_3_ in EtOH, Ti(OPr)_4_, NaBH_4_, 12 h (67% yield); (viii) (Boc)_2_O, TEA, DCM, 0 °C, 2 h (70% yield); (ix) Pd/C, MeOH, H_2_, RT, 4 h (85% yield).

A retro synthetic analysis of compound **C** indicates that the key step would involve the condensation of two key intermediates, i.e., the piperidinyl derivative **9** and the appropriately substituted 6-chloro uracil derivative **11** (Figure 2). The intermediate **9** in turn could be synthesized via functional group manipulations of the spiro derivative **9a**. Preparation of **9a** can be carried out following two strategies e.g. construction of cyclopropyl ring on piperidine moiety (Strategy 1) or vice versa (Strategy 2). While the use of first strategy for the preparation of **9a** has been reported in the literature [11], application of other strategy is not common. Initially, we attempted to synthesize the compound **9a** from *N*-Boc protected piperidone **9b** (PG = Boc) by the reported method [11] based on *C*-alkylation. Surprisingly, introduction of a cyclopropyl group at the position α to the piperidine carbonyl group with 2-chloroethyl dimethyl sulphonium iodide did not work well in our hands. Other methods, e.g., the use of 1,2-dibromoethane in the presence of various bases such as NaH, CH_3_ONa or KO *t*-Bu at different temperatures was also examined but failed to afford the desired product. This prompted us to develop an alternative but more effective method based on strategy 2 (Figure 2) for the synthesis of compound **9** (R = Boc). Apart from our own requirements, we noted that substituted piperidine derivatives containing spirocyclic ring have a wide range of therapeutic effects, namely, as Angiotensin Converting Enzyme (ACE) inhibitors [11], antibacterial agents [12–15], Janus kinase 3 (JAK3) inhibitors [16], Calcitonin-Gene Related Peptide (CGRP) modulators [17] etc. Our synthesis of compound **9** (R = Boc) is shown in Scheme 1.

Commercially available ethyl cyanoacetate and 1,2-dibromoethane was used to construct the appropriately functionalized cyclopropyl ring (step i, Scheme 1) via a conventional C–C bond forming reaction. The reaction proceeded well to afford the functionalized cyclopropane derivative **1** as a result of the formation of two C–C bonds in a single step. Selective reduction of the cyano group of the resulting cyanoester **1** afforded the corresponding amine **2** (step ii, Scheme 1) which on Michael addition to methyl acrylate followed by *N*-protection furnished the diester **4** (steps iii and iv, Scheme 1). The *N*-protection step was necessary to avoid the undesirable participation of the NH group in subsequent steps. A facile base mediated intramolecular cyclization (Dieckmann reaction) [18] of compound **4** provided the key piperidin-4-one ester **5** which on decarboxylation under Krapcho conditions [19] afforded the desired compound **6** (steps v and vi, Scheme 1). Reductive amination of the ketone followed by Boc protection of the resulting amine **7** provided the compound **8** (steps vii and viii, Scheme 1). Finally, deprotection of the secondary amine of compound **8** (step ix, Scheme 1) afforded the target intermediate **9** which was used directly in the next step. It is worth noting that although a shorter synthesis of compound **6** or similar derivative could be achieved starting from *N*-Boc protected piperidin-4-one [11,20], its preparation from ethyl cyclopropanecarboxylate however, requires a longer synthetic route or the tedious preparation of suitable starting materials [21–22].

**Scheme 2:**
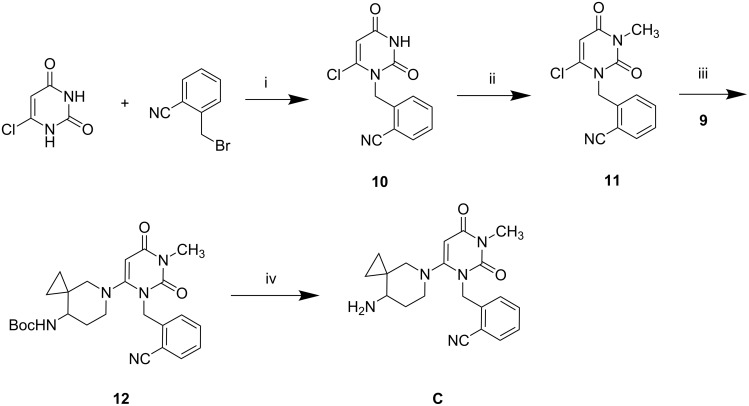
Reagents and conditions: (i) NaH, LiBr, DMF - DMSO, 12 h (55% yield); (ii) NaH, LiBr, CH_3_I, DMF - THF, RT, 12 h (65% yield); (iii) NaHCO_3_, DMSO, 100 °C, 2 h (40% yield); (iv) TFA, THF, RT, 2.5 h (85% yield).

With the piperidinyl intermediate **9** in hand, we then focused our attention on the other intermediate **11**, the preparation of which has been previously documented [23]. The reaction of commercially available 6-chlorouracil with 2-(bromomethyl)benzonitrile in the presence of NaH afforded the *N*-alkylated product **10** which on methylation with methyl iodide provided the target intermediate **11** (steps i and ii, Scheme 2). The chloro compound **11** was then reacted with the amine **9** in the presence of NaHCO_3_ in a sealed tube to give the expected coupled product **12** (step iii, Scheme 2). Finally, *N*-deprotection of compound **12** using trifluoroacetic acid (TFA) gave the target compound **C** as the TFA salt. The ^1^H NMR spectra of compound **C** indicated the presence of a spiro cyclopropyl ring: the four cyclopropyl protons appeared as a series of four multiplets in the region δ 0.15–0.25, 0.30–0.40, 0.50–0.60 and 0.65–0.75, the vinylic proton of pyrimidine-2,4-dione moiety appeared as a singlet at δ 5.36, whilst two doublets at δ 5.11 and 5.23 were indicative of the benzylic methylene hydrogen atoms. This in conjunction with IR bands at 2230 and 1642 cm^−1^ for CN and amide carbonyl group, respectively, characterized compound **C**.

**Figure 3:**
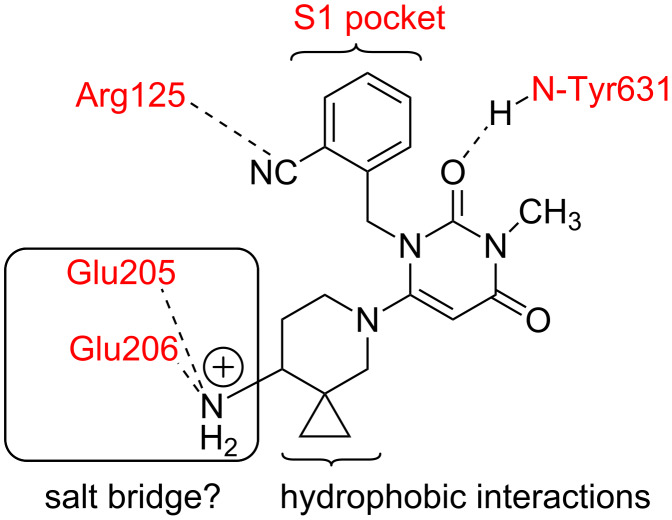
Predicted interactions of compound **C** with DPP-4.

Compound **C** was then tested for its ability to inhibit DPP-4 enzyme in vitro at three concentrations, e.g., 1.0, 5.0 and 10.0 µM [24]. While significant inhibition of DPP-4 was observed at these concentrations, compound **C** however, was found to be less potent than Alogliptin. Based on the interaction [8] of compound **B** with the active site of DPP-4 (Figure 1) it was predicted that (i) the cyanobenzyl group of compound **C** would fill the S1 pocket (formed by V656, Y631, Y662, W659, Y666, and V711) and interact with Arg125, (ii) the carbonyl at C-2 would provide an important hydrogen bond to the backbone NH of Tyr631 (Figure 3). Although the cyclopropyl ring could potentially occupy the nearby empty space to enhance the hydrophobic interactions with DPP-4, the required orientation of the aminopiperidine motif of **C** to form an effective salt bridge to Glu205/Gul206 was probably perturbed. This could be the reason for observed lower potency of **C** in comparison to Alogliptin in vitro. Nevertheless, to gain further insight we plan to conduct Structure-Activity-Relationship (SAR) studies for this class of compound that would eventually help to identify a potential lead possessing favorable pharmacological properties.

## Conclusion

In conclusion, we have developed a route to an aminopiperidine derivative bearing a spirocyclic ring on the piperidine moiety by constructing the cyclopropyl ring prior to assembling the piperidine ring. All steps involved in the present synthesis are simple and based on conventional methods that do not require the use of expensive reagents or catalysts. We have demonstrated the utility of this aminopiperidine derivative for the first time in the preparation of a novel analogue of a potent DPP-4 inhibitor Alogliptin. We believe that the present synthesis could find application in the preparation of diverse aminopiperidine derivatives thereby facilitating the synthesis of novel compounds of potential biological interest.

## Supporting Information

File 1 Experimental procedures and spectral data
