# Supplementary material for: Synthesis of a novel analogue of DPP-4 inhibitor Alogliptin: Introduction of a spirocyclic moiety on the piperidine ring
Source: Beilstein J Org Chem. 2010 Jul 1;6:71. doi: 10.3762/bjoc.6.71 (PMC2919267; doi:10.3762/bjoc.6.71)
Supplement: File 1 — Experimental procedures and spectral data [file Beilstein_J_Org_Chem-06-71-s001.pdf]

Supporting Information

for

Synthesis of a novel analogue of DPP-4 inhibitor Alogliptin: Introduction of a spirocyclic moiety on the piperidine ring

Arumugam Kodimuthali[1,2], Padala Lakshmi Prasunamba[2], Manojit Pal*[1,3]

Address: [1]New Drug Discovery, R&D Center, Matrix Laboratories Ltd, Anrich Industrial Estate, Bollaram, Jinnaram Mandal, Medak District, Andra Pradesh 502 325, India, [2]Department of Chemistry, University College of Science, Osmania University, Hyderabad 500 007, India and [3]present address: Institute of Life Science, University of Hyderabad Campus, Gachibowli, Hyderabad 500 046, Andhra Pradesh, India

E-mail: Manojit Pal - manojitpal@rediffmail.com

*Corresponding author

Experimental procedures and spectral data

General methods: Unless otherwise stated, reactions were performed under a nitrogen atmosphere. Reactions were monitored by thin layer chromatography (TLC) on silica gel coated plates (60 F254), visualizing with ultraviolet light or iodine or $KMnO_4$ solution. Flash chromatography was performed on silica gel (100–200 mesh) using distilled solvents. ^{1}H NMR spectra were determined in $CDCl_3$ or DMSO-d_6 solution at 300 MHz. Proton chemical shifts (δ) are relative to tetramethylsilane (TMS, δ = 0.00) as internal standard and expressed in ppm. Spin multiplicities are given as s (singlet), d (doublet), t (triplet), m (multiplet) and b (broad). Coupling constants (*J*) are given in Hertz. Infrared spectra were recorded on a FT-IR spectrometer. MS spectra were obtained by chemical ionization.

1-Cyanocyclopropanecarboxylic acid ethyl ester (1)

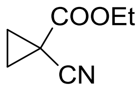

To a stirred solution of ethyl cyanoacetate (3 g, 26.53 mmol) in ethanol (30 mL), was added sodium ethoxide (4.51 g, 66.33 mmol) portionwise at 20 °C over 0.5 h followed by 1,2-dibromoethane (7.47 g, 39.8 mmol). The reaction mixture then was heated and stirred at reflux for 3.5 h. After concentration under vacuum, the residue was diluted with ethyl acetate (30 mL) and neutralized with aq. HCl (10 mL). The organic layer was separated and the aqueous layer extracted with ethyl acetate (2 × 15 mL). The combined organic extracts were dried over anhydrous Na_2SO_4 and concentrated at 40 °C under vacuum. The product was purified by column chromatography using hexane - EtOAc as eluent to yield the title

compound as a colorless liquid (2.58 g, yield 70%); ^{1}H NMR ($CDCl_3$, 300 MHz): δ 1.34 (t, *J* = 7.1 Hz, 3H, *CH*$_3$CH$_2$O-), 1.62 (t, *J* = 3.8 Hz, 2H, *CH*$_2$ c-propyl), 1.67 (t, *J* = 3.8 Hz, 2H, *CH*$_2$ *c*-propyl), 4.25 (q, *J* = 7.1 Hz, 2H, CH$_3$*CH*$_2$O-); MS (CI): 162 (M+Na, 100%); IR (neat, cm^{-1}): 2986, 2249, 1736, 1371, 1311, 1162, 1024, 971, 858, 747; Elemental Analysis found: C, 60.30; H, 6.39; N, 10.24 $C_7H_9NO_2$ Requires C, 60.42; H, 6.52; N, 10.07.

1-Aminomethylcyclopropanecarboxylic acid ethyl ester (2)

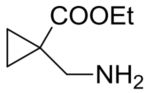

To a stirred solution of compound **1** (2.35 g, 16.9 mmol) in methanol (25 mL), was added 10% Pd on C (25 mg) and the reaction mixture was stirred under hydrogen atmosphere (balloon) at room temperature. On completion of the reaction as indicated by TLC, the mixture was filtered through a celite pad and the filtrate concentrated to yield the title product as a colorless liquid (2.25 g, yield 93%); ^{1}H NMR (DMSO-d_6, 300 MHz): δ 0.8–0.88 (m, 2H, 2 × *CH c*-propyl), 1.0–1.07 (m, 2H, 2 × *CH c*-propyl), 1.16 (t, *J* = 7.1 Hz, 3H, *CH*$_3$CH$_2$O-), 2.65 (s, 2H, *CH*$_2$NH$_2$-methylene), 4.05 (q, *J* = 7.1 Hz, 2H, CH$_3$*CH*$_2$O-); MS (CI): 144.1 (M+1, 100%); Elemental Analysis found: C, 58.51; H, 9.29; N, 9.64 $C_7H_{13}NO_2$ Requires C, 58.72; H, 9.15; N, 9.78.

1-{[Benzyloxycarbonyl-(2-methoxycarbonyl-ethyl)-amino]-methyl}-cyclopropanecarboxylic acid ethyl ester (4)

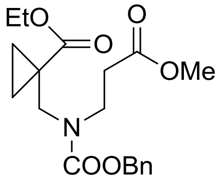

Step 1. To a stirred solution of compound **2** (2.2 g, 15.37 mmol) in THF (25 mL), was added methyl acrylate (1.39 g, 16.14 mmol) dropwise at 0 °C and the mixture slowly warmed to room temperature. After stirring for 3 h at this temperature, no starting material remained as indicated by TLC. The reaction mixture was diluted with cold water (40 mL) and extracted with ethyl acetate (3 × 20 mL). The combined organic layers were washed with water (2 × 50 mL), dried over anhydrous Na_2SO_4 and concentrated at 40 °C under vacuum to give 1-[(2-methoxycarbonyl-ethylamino)-methyl]-cyclopropanecarboxylic acid ethyl ester (**3**) as a liquid (2.61 g, yield 74%). The crude material was used directly for the next reaction.

Step 2. To a stirred solution of compound **3** (2.5 g, 10.91 mmol) in dichloromethane (25 mL), was added triethylamine (1.66 g, 16.37 mmol) at 0 °C followed by the addition of benzyl chloroformate (2.04g, 12.01 mmol).The mixture was stirred at 0–5 °C and the reaction monitored by TLC. After 3 h, the starting material had disappeared and the reaction was quenched in ice water (50 mL). The product was extracted with ethyl acetate (3 x 25 mL). The organic layers were collected, combined, washed with cold water (2 × 25 mL), dried over anhydrous Na_2SO_4 and concentrated at 40 °C under vacuum to give the title compound (3.17 g, yield 80%); ^{1}H-NMR (DMSO-d_6, 300 MHz): δ 0.8–1.0 (m, 2H, 2 × *CH* *c*-propyl), 1.0–1.2 (m, 5H, 2 × *CH* *c*-propyl and $\underline{CH_3}$$CH_2$O-), 2.45–2.55 (m, 2H,

$\underline{CH_2}CO_2Me$), 3.5–3.65 (m, 7H, 4 × *CH* N-methylene and $\underline{CH_3}O$-), 3.95–4.10 (m, 2H, $CH_3\underline{CH_2}O$-), 5.06 (s, 2H, 2 × benzylic methylene), 7.3–7.5 (m, 5H, 5 × *CH* arom); MS (CI): 364.1 (M+1, 100%); Elemental Analysis found: C, 62.53; H, 6.90; N, 4.04 $C_{19}H_{25}NO_6$ Requires C, 62.80; H, 6.93; N, 3.85.

8-Oxo-5-aza-spiro[2.5]octane-5-carboxylic acid benzyl ester (6)

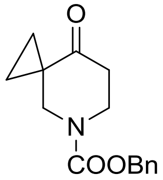

Step 1. To a stirred solution of compound **4** (3.1 g, 8.54 mmol) in DMF (15 mL) at 0 °C, was added 60% sodium hydride in paraffin oil (376 mg, 9.39 mmol) portionwise over a period of 45 min. The reaction mixture was stirred at 0 °C for 30 min followed by stirring at room temperature for 7 h. After completion of the reaction, the reaction was quenched with iced water (50 mL) and the product extracted with ethyl acetate (3 × 25 mL). The combined organic layers were washed with water (4 × 50 mL), dried over anhydrous Na_2SO_4 and concentrated under vacuum to give 8-oxo-5-aza-spiro[2.5]octane-5,7-dicarboxylic acid 5-benzyl ester 7-methyl ester (**5**) as a syrupy liquid (2.33 g, yield 86%). This compound was used in the following step without any further purification or characterization.

Step 2. To a stirred solution of compound **5** (2.2 g, 6.9 mmol) in a mixture of DMSO (15 mL) and water (1 mL), sodium chloride (2.64 g, 45 mmol) was added at room temperature and the reaction mixture was heated and stirred at 110 °C for 7 h. After completion of the reaction, the mixture was cooled to room temperature, quenched with water (50 mL) and extracted with ethyl acetate (3 × 25 mL). The combined organic extracts

were washed with water (4 × 50 mL), dried over anhydrous Na_2SO_4 and concentrated under vacuum to give the title compound as a syrupy liquid (1.35 g, yield 75.5%); ^{1}H NMR (DMSO-d_6, 300 MHz): δ 0.8–0.9 (m, 2H, 2 × *CH* *c*-propyl), 1.0–1.1 (m, 2H, 2 × *CH* *c*-propyl), 2.59 (t, *J* = 6.5 Hz, 2H, 2 × *CH* piperidinyl), 3.6 (s, 2H, 2 × *CH* piperidinyl), 3.75 (t, *J* = 6.5 Hz, 2H, 2 × *CH* piperidinyl), 5.13 (s, 2H, 2 × *CH* benzylic methylene), 7.3–7.4 (m, 5H, 5 × *CH* arom); MS (CI) : 282.1 (M+Na, 100%); Elemental Analysis found: C, 69.71; H, 6.70; N, 4.24 $C_{15}H_{17}NO_3$ Requires C, 69.48; H, 6.61; N, 5.40.

8-Amino-5-aza-spiro[2.5]octane-5-carboxylic acid benzyl ester (7)

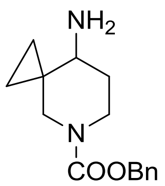

To a stirred solution of compound **6** (1.1 g, 4.24 mmol) in ethanol (8 mL) at 0 °C, was added titanium (IV) isoproxide (2.7 mL, 8.92 mmol) and the reaction mixture was stirred at the same temperature for 2 h. A solution of 10% ethanolic ammonia (25 mL) was added and the reaction mixture stirred at room temperature overnight. The mixture was cooled to 0 °C and sodium borohydride (481 mg, 12.74 mmol) added in portions. The mixture was stirred at 0 °C for 1 h and then at room temperature for 2 h. The mixture was then diluted with aqueous 1.0 N NaOH solution and extracted with dichloromethane (3 × 50 mL). The combined organic extracts were washed with water (2 × 50 mL), dried over anhydrous Na_2SO_4 and concentrated under vacuum to yield the title compound (740 mg, yield 67%); ^{1}H-NMR (DMSO-d_6, 300 MHz): δ 0.3–0.45 (m, 2H, 2 × *CH* *c*-propyl), 0.5–0.65 (m, 2H, 2 × *CH* *c*-propyl), 1.5–1.65 (m, 1H, 1 × *CH* piperidinyl), 1.7–1.9 (m, 1H, 1 × *CH* piperidinyl),

2.78 (t, J = 4.5 Hz, 1H, 1 × *CH* piperidinyl), 3.1–3.16 (m, 1H, 1 × *CH* piperidinyl), 3.3–3.5 (m, 2H, 2 × *CH* piperidinyl), 3.6–3.7 (m, 1H, 1 × *CH* piperidinyl), 5.06 (s, 2H, 2 × *CH* benzylic methylene), 5.9–6.2 (bs, 2H, NH_2), 7.2–7.4 (m, 5H, 5 × *CH* arom); MS (CI): 261.1 (M+1, 100%); Elemental Analysis found: C, 69.11; H, 7.59; N, 10.84 $C_{15}H_{20}N_2O_2$ Requires C, 69.20; H, 7.74; N, 10.76.

8-*tert*-Butoxycarbonylamino-5-aza-spiro[2.5]octane-5-carboxylic acid benzyl ester (8)

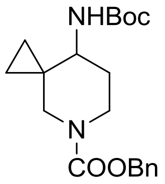

To a stirred solution of compound **7** (700 mg, 2.69 mmol) in DCM (10 ml) at 0 °C, was added triethylamine (544 mg, 5.38 mmol) followed by di-*tert*-butyl dicarbonate (646 mg, 2.96 mmol) and the reaction mixture stirred at 0 °C for 2 h. The reaction was quenched by the addition of water (25 mL) and extracted with ethyl acetate (3 × 20 mL). The combined organic extracts were washed with water (3 × 25 mL), dried over anhydrous Na_2SO_4 and concentrated under vacuum to yield the title compound as a syrupy liquid (678 mg, yield 70%); ^{1}H NMR (DMSO-d_6, 300 MHz): δ 0.19–0.2 (m, 1H, 1 × *CH* *c*-propyl), 0.3–0.4 (m, 2H, 2 × *CH* c-propyl), 0.4–0.5 (m, 1H, 1 × *CH* *c*-propyl), 1.37 (s, 9H, 9 × *CH* *t*-butyl), 1.4–1.5 (m, 1H, 1 × *CH* piperidinyl), 1.6–1.7 (m, 1H, 1 × *CH* piperidinyl), 3.1–3.2 (m, 1H, 1 × *CH* piperidinyl), 3.3–3.4 (m, 2H, 2 × *CH* piperidinyl), 3.45–3.55 (m, 1H, 1 × *CH* piperidinyl), 3.7–3.8 (m, 1H, 1 × *CH* piperidinyl), 5.04 (s, 2H, 2 × *CH* methylene), 6.85 (d, J = 8.9 Hz, 1H, NH), 7.3–7.45 (m, 5H, 5 × *CH* arom); MS (CI): 383.2 (M+Na, 100%);

Elemental Analysis found: C, 66.41; H, 7.79; N, 7.94 $C_{20}H_{28}N_2O_4$ Requires C, 66.64; H, 7.83; N, 7.77.

2-(6-Chloro-2,4-dioxo-3,4-dihydro-2*H*-pyrimidin-1-ylmethyl)-benzonitrile (10)

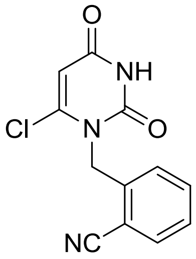

To a stirred solution of 6-chlorouracil (20 g, 13.6 mmol) in a mixture of DMF – DMSO (6:1, 60 mL) at 0 °C was added sodium hydride (60%, 655 mg, 16.3 mmol) portionwise and the mixture was stirred for 30 min. LiBr (1.13 g, 13.6 mmol) was added at the same temperature and the mixture stirred for another 15 min, followed by the addition of a solution of α-bromo-*o*-tolunitrile (2.67 g, 13.6 mmol) in DMF (4.0 mL). The reaction mixture was stirred 1 h at 0 °C and then for 12 h at room temperature. After concentration under vacuum, the residue was diluted with water (100 mL) and the precipitated solid filtered and dried. The solid was treated with EtOAc - $CHCl_3$ and the mixture heated at 60 °C for 15 min. The mixture was then cooled to 0 °C, stirred for 1 h, filtered and dried to yield the title compound (1.94 g, 55%); ^1H NMR (DMSO-d_6, 300 MHz): δ 5.3 (s, 2H), 6.06 (s, 1H), 7.38 (d, J = 7.9 Hz, 1H), 7.52 (t, J = 7.6 Hz, 1H), 7.68 (t, J = 7.6 Hz, 1H), 7.88 (d, J = 7.6 Hz, 1H), 11.78 (s, 1H); MS (CI): 262 (M+1, 100%); Elemental Analysis found: C, 55.21; H, 3.09; N, 15.84 $C_{12}H_8ClN_3O_2$ Requires C, 55.08; H, 3.08; N, 16.06.

2-(6-Chloro-3-methyl-2,4-dioxo-3,4-dihydro-2H-pyrimidin-1-ylmethyl)-benzonitrile (11)

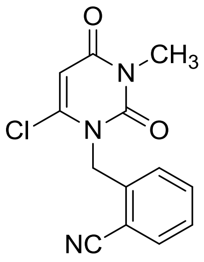

To a stirred solution of **10** (1 g, 3.8 mmol) in DMF - THF (1:1, 30 mL) at 0 °C, was added sodium hydride (60%, 168 mg, 4.0 mmol) portionwise, followed by LiBr (200 mg, 2.4 mmol) maintaining the temperature at 0 °C. The mixture was stirred at room temperature for 30 min and then cooled to 0 °C. Iodomethane (0.54 mL, 7.6 mmol) was added and the mixture stirred at room temperature for 12 h under sealed conditions. The reaction mixture was concentrated under vacuum and the residue diluted with dichloromethane (30 mL). The organic layer was separated, washed with water (3 × 50 mL), dried over anhydrous Na_2SO_4 and concentrated under vacuum. The crude product was purified by column chromatography using heptane - CH_2Cl_2 as eluent to give the desired compound (686 mg, yield 65%); ^1H NMR (DMSO-d_6, 300 MHz): δ 3.18 (s, 3H), 5.38 (s, 2H), 6.21 (s, 1H), 7.41 (d, J = 7.8 Hz, 1H), 7.51 (t, J = 7.5 Hz, 1H), 7.71 (t, J = 7.5 Hz, 1H), 7.89 (d, J = 7.6 Hz, 1H); MS (CI): 276.0 (M+1, 100%); Elemental Analysis found: C, 56.41; H, 3.69; N, 15.53 $C_{13}H_{10}ClN_3O_2$ Requires C, 56.64; H, 3.66; N, 15.24.

***tert*-Butyl 5-(3-(2-cyanobenzyl)-1-methyl-2,6-dioxo-1,2,3,6-tetrahydropyrimidin-4-yl)-5-azaspiro[2.5]octan-8-ylcarbamate (12)**

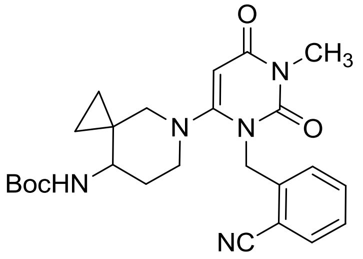

Step 1. To a stirred solution of compound **8** (600 mg, 1.67 mmol) in methanol (10 mL), was added 10% Pd/C (30 mg) and the reaction mixture was stirred under a hydrogen atmosphere (balloon) at room temperature. After 5 h, the starting material had disappeared as indicated by TLC and the reaction mixture was filtered through a celite pad. The pad was washed with methanol (10 mL). The combined filtrate and washings was concentrated under vacuum to give (5-aza-spiro[2.5]oct-8-yl)-carbamic acid tert-butyl ester (**9**) as syrupy liquid (320 mg, 85%). The compound was directly used in the following step.

Step 2. A mixture of compound **11** (200 mg, 0.73 mmol), **9** (164 mg, 0.73 mmol) and sodium bicarbonate (122 mg, 1.45 mmol) in DMSO (4 mL) were placed in a sealed tube and heated at 100 °C for 2 h. The reaction mixture was filtered through a celite pad and the filtrate concentrated under vacuum. The residue was diluted with dichloromethane (75 mL) and washed with water (3 × 30 mL). The organic layer was dried over anhydrous Na_2SO_4 and concentrated. The residue was purified by column chromatography using MeOH - $CHCl_3$ as eluent to give the desired product (135 mg, yield 40%); ^{1}H NMR (DMSO-d_6, 300 MHz): δ 0.10–0.15 (m, 2H, 2 × *CH* c-propyl), 0.2–0.3 (m, 1H, 1 × *CH c*-propyl), 0.4–0.5 (m, 1H, 1 × *CH c*-propyl), 1.36 (s, 9H, 9 × *CH tert*-butyl), 1.5–1.7 (m, 1H, 1 × *CH* piperidinyl), 1.7–1.85 (m, 1H, 1 × *CH* piperidinyl), 2.6–2.8 (m, 2H, 2 × *CH* piperidinyl),

2.85–3.0 (m, 1H, 1 × *CH* piperidinyl), 3.09–3.26 (m, 4H, 1 × *CH* piperidinyl and NCH_3), 3.3–3.5 (m, 1H, 1 × *CH* piperidinyl), 5.15 (s, 2H, 2 × *CH* methylene), 5.37 (s, 1H, 1 × *CH* vinyl), 6.83 (bs, 1H), 7.20 (d, *J* = 7.9 Hz, 1H, 1 × *CH* arom), 7.46 (t, *J* = 7.6 Hz, 1H, 1 × *CH* arom), 7.65 (t, *J* = 7.6 Hz, 1H, 1 × *CH* arom), 7.84 (d, *J* = 7.6 Hz, 1H, 1 × *CH* arom) ; MS (CI): 466.2 (M+1, 100%); IR (KBr, cm^{-1}): 3348 (NH), 2225 (CN), 1718 (CO), 1667 (CO), 1524, 1442, 1303, 1164, 1023, 766; Elemental Analysis found: C, 64.31; H, 6.69; N, 15.13 $C_{25}H_{31}N_5O_4$ Requires C, 64.50; H, 6.71; N, 15.04.

2-[6-(8-Amino-5-aza-spiro[2.5]oct-5-yl)-3-methyl-2,4-dioxo-3,4-dihydro-2*H*-pyrimidin-1-ylmethyl]-benzonitrile·TFA (C)

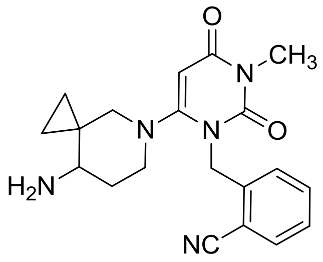

To a stirred solution of **12** (100 mg, 0.21 mmol) in THF (5 ml), was added TFA (49 mg, 0.42 mmol) and the reaction mixture was stirred for 2.5 h at room temperature. The mixture was concentrated under vacuum, the residue washed with diisopropyl ether (2 × 10 mL) and dried under vacuum to give the title compound as an off-white solid (87 mg, yield 85%); mp > 300 ºC; ^1H NMR (DMSO-d_6, 300 MHz): δ 0.15–0.25 (m, 1H, 1 × *CH* *c*-propyl), 0.30–0.40 (m, 1H, 1 × *CH* *c*-propyl), 0.50–0.60 (m, 1H, 1 × *CH* *c*-propyl), 0.65–0.75 (m, 1H, 1 × *CH* *c*-propyl), 1.70–1.85 (m, 1H, 1 × *CH* piperidinyl), 1.95–2.10 (m, 1H, 1 × *CH* piperidinyl), 2.25–2.40 (m, 1H, 1 × *CH* piperidinyl), 2.80–2.95 (m, 1H, 1 × *CH* piperidinyl), 3.0–3.30 (m, 6H, NCH_3 and 3 × *CH* piperidinyl), 5.11 (d, *J* = 16.0 Hz, 1H, 1 × *CH*

methylene), 5.23 (d, J = 16.3 Hz, 1H, 1 × CH methylene), 5.36 (s, 1H, 1 × CH vinyl), 7.22 (d, J = 7.8 Hz, 1H, 1 × CH arom), 7.47 (t, J = 7.6 Hz, 1H, 1 × CH arom), 7.65 (t, J = 7.6 Hz, 1H, 1 × CH arom), 7.85 (d, J = 7.7 Hz, 1H, 1 × CH arom), 7.90-8.10 (bs, 3H, NH_3^+); MS (CI): 366.2 (M+1, 100%); IR (KBr, cm^{-1}): 3434 (bs, NH), 3072, 2230 (CN), 1690 (CO of TFA salt), 1642 (CO_{amide}), 1542, 1470, 1437, 1201, 1132, 763; Elemental Analysis found: C, 55.31; H, 5.09; N, 14.30 $C_{22}H_{24}F_3N_5O_4$ Requires C, 55.11; H, 5.05; N, 14.61.

Pharmacological method: Determination of DPP-4 inhibition in vitro

Assay conditions: Assay buffer used for DPP-4 inhibition studies was 25 mM TRIS-HCl, 140 mM NaCl, 10 mM KCl, 0.01% BSA. All working stocks of compound **C**, DPP-4 enzyme and substrate were prepared in assay buffer. Inhibition studies were performed by incubating different concentrations of compound **C** with 10 ng/well of human DPP-4 for 15 min at 30 ºC. Enzyme activity was initiated by 50 μM of substrate Gly-Pro-AMC. Fluorescence was measured after 30 min incubation at 30 ºC. Calculations for DPP-4 enzyme percent inhibition were carried out using the formula shown below:

% DPP-4 inhibition = 100-(RFU of test - RFU of blank) / (RFU of control - RFU of blank)* × 100

(RFU: relative fluorescence units)

LAF-237 was used as a reference compound in this experiment and gave an IC_{50} of 5.79 ± 1.07 nM, which was close to the reported literature value of 3.5 ± 1.2 nM (see for example, Villhauer, E. B.; Brinkman, J. A.; Naderi, G. B.; Burkey, B. F.; Dunning, B. E.; Prasad, K.; Mangold, B. L.; Russel, M. E. and Hughes, T. E. *J. Med. Chem.* **2003,** *46*, 2774–2789).
